# Microbiome interventions combined with artificial humic acid treatments for restoring soil bacterial diversity

**DOI:** 10.1093/sumbio/qvag012

**Published:** 2026-03-18

**Authors:** Wisnu Adi Wicaksono, Samuel Bickel, Julia Peissl, Nader Marzban, Daniel Hoefle, Ahmed Abdelfattah, Markus Antonietti, Gabriele Berg

**Affiliations:** Institute of Environmental Biotechnology, Graz University of Technology, Graz 8010, Austria; Institute of Environmental Biotechnology, Graz University of Technology, Graz 8010, Austria; Institute of Environmental Biotechnology, Graz University of Technology, Graz 8010, Austria; Department of Microbiome Biotechnology, Leibniz Institute for Agricultural Engineering and Bioeconomy (ATB), Potsdam 14469, Germany; Department of Microbiome Biotechnology, Leibniz Institute for Agricultural Engineering and Bioeconomy (ATB), Potsdam 14469, Germany; Institute for Biochemistry and Biology, University of Potsdam, Potsdam 14476, Germany; Department of Microbiome Biotechnology, Leibniz Institute for Agricultural Engineering and Bioeconomy (ATB), Potsdam 14469, Germany; Department of Colloid Chemistry, Max Planck Institute of Colloids and Interfaces, Potsdam 14476, Germany; Institute of Environmental Biotechnology, Graz University of Technology, Graz 8010, Austria; Department of Microbiome Biotechnology, Leibniz Institute for Agricultural Engineering and Bioeconomy (ATB), Potsdam 14469, Germany; Institute for Biochemistry and Biology, University of Potsdam, Potsdam 14476, Germany; Department of Colloid Chemistry, Max Planck Institute of Colloids and Interfaces, Potsdam 14476, Germany

**Keywords:** soil, soil microbes, amplicon sequencing, environmental microbiology, microbial diversity

## Abstract

Soil health is under threat worldwide and technologies for soil restoration are urgently needed. Here we study the effect of artificial humic acids (A-HA) and soil transplants fo one restoration of depleted soil microbiomes. We used a controlled microcosm experiment with gradients of microbial diversity, with and without A-HA, across three soil types. Microbial abundance, diversity, and composition were assessed using qPCR and 16S rRNA gene amplicon sequencing, complemented by metabolomic profiling of water-extractable compounds and the growth characterization of bacterial isolates. A-HA treatment had a stronger effect on bacterial richness and community structure in degraded than in the original soil. Soil microbiome transplants could partially regenerate microbial abundances and increased bacterial richness and diversity in the degraded soils. Interestingly, the combination of A-HAs with addition of 10% soil transplants yielded the best restoration effect. The effect of individual as well as combined treatments strongly depended on the composition of the native soil microbiome. From a mechanistic point of view, A-HA treatment inhibited fast-growing bacteria, which allowed slow-growing bacteria to recover. By combined treatment, depending on the soil type and its native soil microbiome, we can synergistically restore the soil microbiome to resemble its original composition.

**Sustainability statement**

This study supports **Sustainable Development Goal 15 (Life on Land)** by contributing to the restoration and sustainable management of terrestrial ecosystems through improved soil health. By combining soil transplants and humic acid amendments, the research addresses environmental challenges that disrupt soil microbiomes and degrade soil. This sustainable strategy enhanced soil microbial diversity that potentially led to improved soil functionality, contributing to ecosystem resilience and countering biodiversity loss. By restoring soil microbiomes, this research advances nature-based solutions that sustain healthy ecosystems and support land restoration.

## Introduction

Soil health is essential for the functioning of all terrestrial ecosystems, including biogeochemical cycles, water storage and supply, climate regulation, and biodiversity conservation (Lehmann et al. 2020, Beattie et al. 2025). Globally, soil is under pressure from various anthropogenic activities, increases in human population size, intense agriculture, and competing land uses (Rillig et al. 2019). Approximately 33% of our global soils are already degraded (FAO 2015), and over 60% of European soils are unhealthy (EU 2023). Certain agricultural practices i.e. intense management (Tsiafouli et al. 2015), high pesticide usage (Sim et al. 2022, Ni et al. 2025), and microplastics (Han et al. 2024), lead to a decline in soil microbial diversity and functionality, which hinders their capacity to support essential soil functions. Restoring soil health is crucial for ensuring food security and ecosystem health but this needs clear definitions and knowledge-based intervention strategies.

The definition of soil health is widely discussed and comprises diverse chemical, physical, and biological indicators. Soil microbial diversity has emerged as a valuable indicator of soil health (Fierer et al. 2021). The soil microbiome is the living part of soil, and the key to various ecological services, including nutrient cycling, pest control, and soil formation and modulation, as well as a driver of plant diversity and productivity (Van Der Heijden et al. 2008, Philippot et al. 2024). The soil microbiome, plant microbiome, and plant health are also strongly interconnected (Banerjee and Van Der Heijden 2023). Evidence for the positive relationship between soil bacterial diversity and plant productivity has been demonstrated (Saleem et al. 2019, Chen et al. 2020).

Knowledge-based intervention strategies could be applied to soil microbiome restoration. This can be achieved directly by applying (i) microbiome transplants, (ii) microbes with beneficial properties, (iii) microbiota-active metabolites, and (iv) indirectly by changing abiotic factors (Berg et al. 2020). Microbiome transplants, which involves transferring soil from a healthy ecosystem to a degraded site, represents a promising approach for directly restoring soil microbiome diversity and functionality (King et al. 2023, Pedrinho et al. 2024). However, studies showed that the efficacy of soil microbiome restoration strongly depends on soil type, and certain soil functions cannot fully recovered, indicating limitations associated with microbial community transplants (Calderón et al. 2017). Another direct strategy involves the application of microbiota-derived active metabolites (Olimi et al. 2025) or organic amendments such as composts, and biochar (Bonanomi et al. 2018). This approach can stimulate indigenous beneficial microbial communities, enhance microbial biomass, and promote key soil functions essential for soil health and fertility. In addition, soil microbiome restoration can be achieved indirectly by modifying abiotic factors such as pH, nutrient availability, moisture regimes, and soil physical structure (Bonanomi et al. 2018). Thus, combining multiple strategies is critical for enhancing soil microbiome restoration by creating favorable conditions for microbial activity, stability, and long-term functionality.

Humic acids are widely recognized as important regulators of soil fertility and soil organic matter dynamics. There is increasing evidence that humic acid-based products support sustainable agriculture and enhance soil health (Ampong et al. 2022). While natural humic acids from lignite or peat have been used for decades, recent advances in controlling the chemistry of humification allowed the rapid and sustainable synthesis of artificial humic acids (A-HA) from biomass and waste through biotic composting and abiotic processes (Yang et al. 2019). A-HAs can be used in agricultural applications to improve the physical and chemical properties of soil, leading to improved soil fertility, crop growth, and quality (reviewed in Ampong et al. 2022, Yang et al. 2023). The positive effects of A-HA on plant and soil health have already been described (Li et al. 2019, Ghaslani et al. 2024, Hoefle et al. 2024, Rui et al. 2024) but their impact on soil microbial communities remains underexplored (Lumactud et al. 2022), indicating a need for further investigation and understanding of the effects of A-HA on the soil microbiome. Our hypothesis was that a combined soil transplant and A-HA treatment, which were rarely studied, can synergistically shape and restore the soil microbiome in the degraded soil.

This study investigates the potential application of a combined approach of A-HA amendment and soil transplant for the restoration of depleted soil microbiomes. We are using a comprehensive approach that includes culture-dependent growth assays and culture-independent methods, such as qPCR and amplicon sequencing, to address the following research questions: (1) Does soil transplantation enhance soil bacterial diversity? (2) How does soil transplantation interact with the addition of humic acid? (3) Is there a consistent response observed across different soils? Three distinct soil types originated from urban gardening, agriculture, and horticulture were evaluated to determine if the soil and its microbiome respond differently to the treatments and to assess how the restoration of the soil microbiome is influenced by the native microbiome and the different soil traits.

## Materials and methods

### Soil collection and humic acid preparation

In this study, we aimed to analyze the impact of soil transplant and amendment of humic acid on bacterial community composition of three soils with different land management histories. For the 1st experiment, residential garden soil, hereafter referred to as soil RG, was obtained in an urban region of Graz (Austria; 47°4′ 14″ N 15° 25′ 20″ E). For the 2nd experiment, soil samples were collected from an extensive grassland area located in Graz at 47°04′51″ N, 15°27′24″ E and an organically managed apple orchard at 47°07′44″ N, 15°46′50″ E, hereafter referred to as soil EG and soil AO, respectively. Soil properties are detailed in Supplementary Table S1. The top layer of each soil, measuring 10–20 cm deep, was collected and sieved through a 3 mm sieve to remove rocks and debris. To calculate the necessary water saturation for each soil sample, soil water content was evaluated by weighing the soil before and after 48 h at 105°C and adjusted to 25% soil water content.

Artificial humic acid (A-HA) used in this study was synthesized using the hydrothermal humification technique as described in our previous work (Marzban et al. 2024). Briefly, 600 g of digestate slurry with a solids content of 8.3% (wt, dry basis) and 37.9 g of KOH (corresponding to 4.5 equivalents of the total carbohydrate content) were placed in a 1-L high-pressure batch reactor (Parr Instrument Company, Series 4520, Moline, IL, USA). The alkaline condition, maintained by KOH, facilitated the conversion of biomass into artificial humic substances and defined the process as hydrothermal fulvification (Marzban 2024). The reactor was heated to 240°C and maintained for 4 h under autogenous pressure. After cooling, the reaction mixture was filtered using 5–8 µm flat filter paper (ROTH Type 113A-110) to separate solids from the liquid. Artificial humic acid was then extracted from the liquid phase by acidification with 32% HCl to adjust the pH to 1.0, followed by centrifugation. The resulting A-HA was dried at temperatures below 60°C for 48 h, following the established method for processing the hydrothermal process liquid. The final A-HA yield was 37.5%, based on the dry mass of the digestate. The resulting A-HA had a purity exceeding 96% and exhibited chemical, elemental, and functional similarities to natural humic substances. The product was characterized by a wide range of techniques, as reported previously (Tkachenko et al. 2023, Marzban et al. 2024, Volikov et al. 2024). The dried A-HA was stored in a desiccator until used in the soil restoration experiment.

### Experimental design and sampling

**Figure 1:**
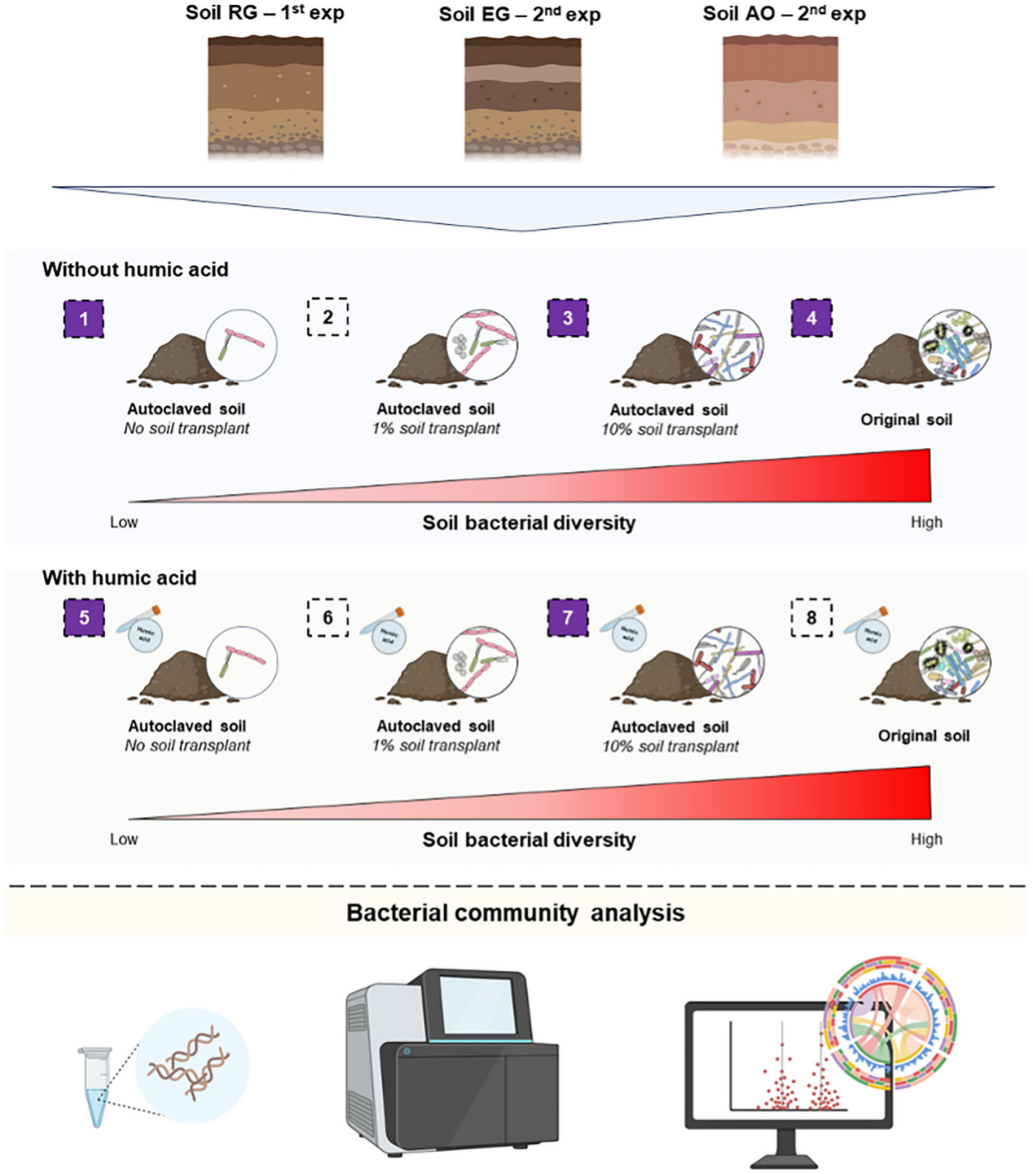
Summary of experimental design. In this study, three distinct soil types were utilized. A gradient of soil bacterial diversity was established through soil transplantation, following the sequence: autoclaved soil, autoclaved soil supplemented with 1% soil transplant, autoclaved soil supplemented with 10% soil transplant, and the original soils. These treatments were performed both with and without the addition of humic acid. The treatment that was colored purple was only done in the 2nd experiment.

The experimental configuration is summarized in Fig. 1. Each soil sample was split into two portions: one portion was identified as the source soil (original) and kept at 4°C for up to one week until use, and the other portion was treated by autoclaving, defined as a degraded soil. Autoclaving was chosen as the soil sterilization method due to existing evidence suggesting that the selection of sterilization technique has minimal impact on subsequent microbial recolonization. Consequently, autoclaving is considered a reliable, reproducible, and cost-effective method for achieving effective biotic clearance (King et al. 2024). These soils were autoclaved three times, with a one-day interval between each cycle, at 121°C for 45 min. Afterwards, the moisture levels of the original and degraded soils were adjusted to a common value.

We hypothesized that applying soil transplants and A-HA would enhance bacterial diversity in soils with low levels of bacterial diversity. This was achieved by creating a gradient of soil bacterial diversity by adding varying concentrations of the source soil (1% and 10% v/v) to the degraded soil with and without A-HA. Therefore, for the first experiment, a total of eight treatments were prepared, i.e. (1) original soil, (2) original soil with A-HA, (3) degraded soil, (4) degraded soil with A-HA, (5) degraded soil with 1% soil transplant, (6) degraded soil with 1% soil transplant and A-HA, (7) degraded soil with 10% soil transplant, and (8) degraded soil with 10% soil transplant and A-HA (Fig. 1). Artificial humic acid was weighed (450 mg HA per kg soil), dissolved in 5 mL of 0.05 mol/L NaOH, following the procedure previously described (Yuan et al. 2022). For each of the soil samples, A-HA was uniformly applied to 250 g of soil in each plastic container to establish the microcosm experiment. The second experiment was carried out based on the results of the first experiment (see “Results” section). Following our observation that soil transplantation and the application of humic acid increased bacterial diversity and altered community composition, the second experiment was conducted using two distinct soil types to assess whether humic acid treatment consistently influences bacterial community structure across different soil environments. Therefore, five treatments, i.e. (1) original soil, (2) degraded soil, (3) degraded soil with A-HA, (4) degraded soil with 10% transplant, and (5) degraded soil with 10% soil transplant and A-HA. The second experiment was done using soil EG and AO. The soil microcosms were randomized and placed in the greenhouse under controlled growing conditions, at a temperature of 23°C, 60% humidity, and 16:8 h light/dark conditions for 10 days.

Each soil sample (approx. 500 mg) underwent processing using the FastDNA SPIN Kit for Soil and the FastPrep Instrument (MP Biomedicals, Santa Ana, CA, USA) to extract the total DNA following the manufacturer’s guidelines. The quality and quantity of the extracted DNA assessed using a NanoDrop 2000 device (Thermo Fisher Scientific; Wilmington, DE, USA). The DNA samples were then stored at -20°C for future processing.

### Quantification of bacteria using qPCR

We used quantitative real-time PCR (qPCR) with SYBR Green fluorescence to determine the bacterial abundance in the soil samples (measured as copies of marker genes per gram of soil). We employed specific primer sets to determine the absolute abundance of total bacteria (primer 515f-806r—Caporaso et al. 2011), *Actinobacteria* (primer Actino243f-Actino513r—Heuer et al. 1997), and *Gammaproteobacteria* (Gamma395f-Gamma871r—Mühling et al. 2008). The qPCR reaction consisted of 1 μL of extracted DNA, 5 μL of KAPA SYBR® FAST qPCR Master Mix (2X) (KAPA Biosystem, USA), 0.2 mM of each primer, and PCR-grade water. The fluorescence quantification process was performed using the Rotor-Gene 6000 real-time rotary analyzer (Corbett Research, Sydney, Australia) with initial denaturing at 95°C for 10 min, followed by 40 cycles of denaturing at 95°C for 30 s, annealing at 50°C (for total bacteria) or 62°C (for *Actinobacteria*) or 56°C (for *Gammaproteobacteria*) for 30 s, and extension at 72°C for 30 s, and a final melting curve.

### 16S rRNA gene amplicon sequencing and bioinformatic analysis

16S rRNA gene amplicon sequencing was conducted by amplifying the 16S rRNA gene V4 hypervariable region from total DNA using the primer pair 515f-806r (Caporaso et al. 2011). Each primer included sample-specific barcodes for demultiplexing, following the Earth Microbiome Project protocol (https://earthmicrobiome.org/). PCR reactions were conducted in a 25 μL volume, using the 2 × KAPA Taq Ready Mix (Kapa Biosystems, USA), 0.2 mM of each primer, PCR-grade water, and 1 μL of undiluted DNA. Two technical replicates were utilized for each sample. PCR reactions were performed in Whatman Biometra® Tpersonal thermocycler (Biometra GmbH, Göttingen, Germany) with 35 cycles at 94°C for denaturation for 45 s, 54°C annealing for 60 s, and 72°C elongation for 90 s. The amplified PCR products were confirmed by visualizing them on agarose gels and purifying them using the Wizard® SV Gel and PCR Clean-Up kit (Promega, Madison, WI, USA). The barcoded amplicons were sequenced on an Illumina NovaSeq instrument (2 × 250 bp paired-end reads) by the sequencing provider Novogene (Cambridge, UK). The amplicon sequences have been deposited in the European Nucleotide Archive under the project number PRJEB94360.

The paired-end raw reads underwent demultiplexing and primer sequences were removed using cutadapt (Martin 2011). The demultiplexed reads were then processed through the open-source QIIME2 pipeline (Bolyen et al. 2019). The DADA2 algorithm (Callahan et al. 2016) was utilized for quality filtering, denoising, and removal of chimeric sequences, resulting in representative amplicon sequence variants (ASVs) and a feature table. ASVs were further classified using the vsearch algorithm (Rognes et al. 2016) against the SILVA v138 database (Quast et al. 2012).

### Profiling of water-extractable metabolites

Analysis of the soil samples was conducted using high-performance liquid chromatography (HPLC) coupled with mass spectrometry (MS). For the first experiment, we analyzed only the effect of autoclaving the soil, while all treatments were analyzed for the second experiment. The soil samples were suspended in deionized water using 5 g of soil in 7 mL water. The samples were placed on a shaker for 2 h at 200 rpm before centrifugation at 10 000 rpm for 10 min. The supernatant was transferred into 15 mL Falcon tubes and stored at −20°C. The extracts were analyzed using the hybrid quadrupole orbitrap mass spectrometer HPLC Ultimate 3000 (Q Exactive; Thermo Scientific, Bremen, Germany). The column Waters Atlantis dC18 (Phenomenex, Aschaffenburg, Germany) with 3 μm particle size and dimensions 2.1 mm × 100 mm was used. The solvents were formic acid in acetonitrile (0.2%, v/v), and formic acid in water (0.2%, v/v). Throughout the measurements, temperature was maintained at 25°C with a flow rate of 0.3 mL/min. For each sample, the starting column conditions were 5% acetonitrile for 3 min. Gradient elution conditions over 35 min were 5% to 80% acetonitrile. Then the solvent was set to 5% acetonitrile for the measurement. Sample analysis was performed with negative and positive ion detection. The electrospray ionization conditions were 350°C capillary temperature at 3.2 kV spray voltage. The scans for metabolites were in the range of 100–1500 m/z with a resolution of 70 000 full width at half maximum and full MS/dd-MS2 mode was used. The spectra were analyzed using Compound Discoverer 3.3 (Thermo Fisher Scientific) and blank measurement (water) was subtracted from each compounds’ peak area.

### Isolation, growth measurement, and identification of culturable bacterial strains

For the bacterial isolation process, 5 g of each original soil (soil RG, EG, and AO) was mixed with 10 mL of a 0.9% NaCl solution. This mixture was utilized to cultivate bacteria on various media, including nutrient broth II agar (NA) media (SIFIN, Berlin, Germany), Reasoner’s 2A (R2A) (Carl Roth GmbH + Co. KG; Karlsruhe, Germany), and agar plates prepared from soil-extracted liquid (200 g of autoclaved soil per 500 mL of water with 1.5% agar). A total of 218 strains were successfully isolated from the three soil samples. The colonies were then transferred to 96-well plates containing Nutrient Broth II and 30% glycerol for long-term storage. These cultures were stored at −70°C at the Institute of Environmental Biotechnology at Graz University of Technology in Graz, Austria.

Prior to the growth measurement of the bacterial isolates, 200 µL of Tryptic Soy Broth (TSB, Sigma-Aldrich, USA) medium was inoculated with 10 µL of the bacterial glycerol stock and incubated for 16 h at 30°C. From these overnight cultures, 10 µL of the bacterial cultures were transferred into a new 200 µL medium and incubated at 30°C for an additional 2 h. After this incubation period, another 10 µL was transferred into a new 96-well plate containing 200 µL of TSB medium. The optical density at 600 nm (OD_600_) was recorded over a 24-h period using the Tecan microplate reader Infinite^®^ 200 PRO (Tecan Austria GmbH, Grödig, Austria), with measurements taken every 20 min within a temperature range of 24–26°C. The 96-well plate measurements were performed in triplicates to obtain the average growth rate of each isolate. Using the observed OD_600_ values, the maximum growth rate (k_max_) was determined to calculate the doubling time (t_D_ = ln(2)/k_max_). Only those isolates exhibiting growth exceeding the negative control (medium without inoculation) were included in the analysis.

A total of 188 randomly selected bacterial isolates were identified by analyzing the sequences of their 16S rRNA gene fragments, utilizing the primer set 27F and 1492R. Bacterial DNA was isolated using a rapid DNA extraction protocol involving high-temperature treatment for cell lysis. A bacterial colony was transferred to a 1.5 mL Eppendorf tube containing 100 µL of PCR-grade water. The sample was then stored at −70°C for 15 min, followed by heating at 100°C for 5 min. Subsequently, the sample was centrifuged, and the supernatant was collected for PCR analysis. Polymerase chain reactions (PCRs) were conducted using a Mastercycler^®^ nexus thermocycler (Eppendorf, Hamburg-Nord, Germany). The amplified 16S rRNA genes were Sanger sequenced by the commercial sequencing service Microsynth (Vienna, Austria). Following sequencing, manual quality filtering was done to eliminate ambiguous sequences utilizing BioEdit. Our objective was to investigate the origins of ASVs that exhibited differential abundance in humic acid-treated soils, focusing on both fast—those with shorter doubling times—and slow-growing bacterial populations. To achieve this, we aligned the differentially abundant ASVs identified through DeSeq2 with the 16S rRNA sequences of culturable bacterial strains for which we measured doubling times, as previously described. We determined that ASV matches based on successful mapping with 100% coverage and at least 97% sequence identity against reference sequences obtained from isolated strains with known doubling times.

### Statistical analysis

Statistical analysis and data visualization were performed using R version 4.1.2 (Core Team 2013). To evaluate significant differences in microbial gene copy numbers, the Kruskal–Wallis test was used, followed by post-hoc Dunn’s tests for multiple comparisons. The microbial community analysis was based on ASV tables and taxonomic classifications, processed with the phyloseq v1.38.0 (McMurdie and Holmes 2013). The dataset was normalized by randomly selecting subsets of sequences from the lowest read counts (soil RG–7361 reads, soil EG–23 561 reads, and soil AO–147 434 reads), and bar plots were generated to depict the taxonomic composition. Changes in alpha diversity based on the number of bacterial ASV and Shannon diversity index (H’) from the rarefied dataset were assessed using the Kruskal–Wallis test, with post-hoc Dunn’s tests, applying a significance level of *P* < 0.05. Non-metric Bray–Curtis dissimilarity matrices were created and analyzed using permutational multivariate analysis of variance (Adonis, with 999 permutations) to explore the effects of various factors, such as soil autoclaving, soil transplant, and humic acid amendment, on microbial community structures. Moreover, pairwise Bray–Curtis dissimilarities were calculated to quantify variation in soil microbial community composition among samples across different treatments. A two-dimensional non-metric multidimensional scaling (NMDS) plot was constructed to visualize the distance matrices, and hierarchical clustering was conducted using the Bray–Curtis dissimilarity matrix. The DeSeq2 method was utilized to identify differentially abundant ASVs in humic-treated soils, with an adjusted *P*-value threshold set at less than 0.05.

For the metabolome data, we determined the number of compounds and the number of unique compounds for the different treatments. For assessing changes in the mixtures, only the compounds that were present in all samples were considered. The peak area values were log-transformed before computing z-scores for different compounds. The z-score values were used for clustering, PCA, and the calculation of Euclidean distances concerning the original soils’ mixtures. Furthermore, pairwise Euclidean dissimilarities were calculated to assess variation in metabolic composition among samples subjected to different treatments. To investigate if compounds were, on average, enriched or depleted through HA treatment, we computed the log response ratio and standard errors (Pustejovsky 2015).

## Results

### Experiment 1–soil transplant and humic acid amendment led to alterations in bacterial diversity and community structures

**Figure 2:**
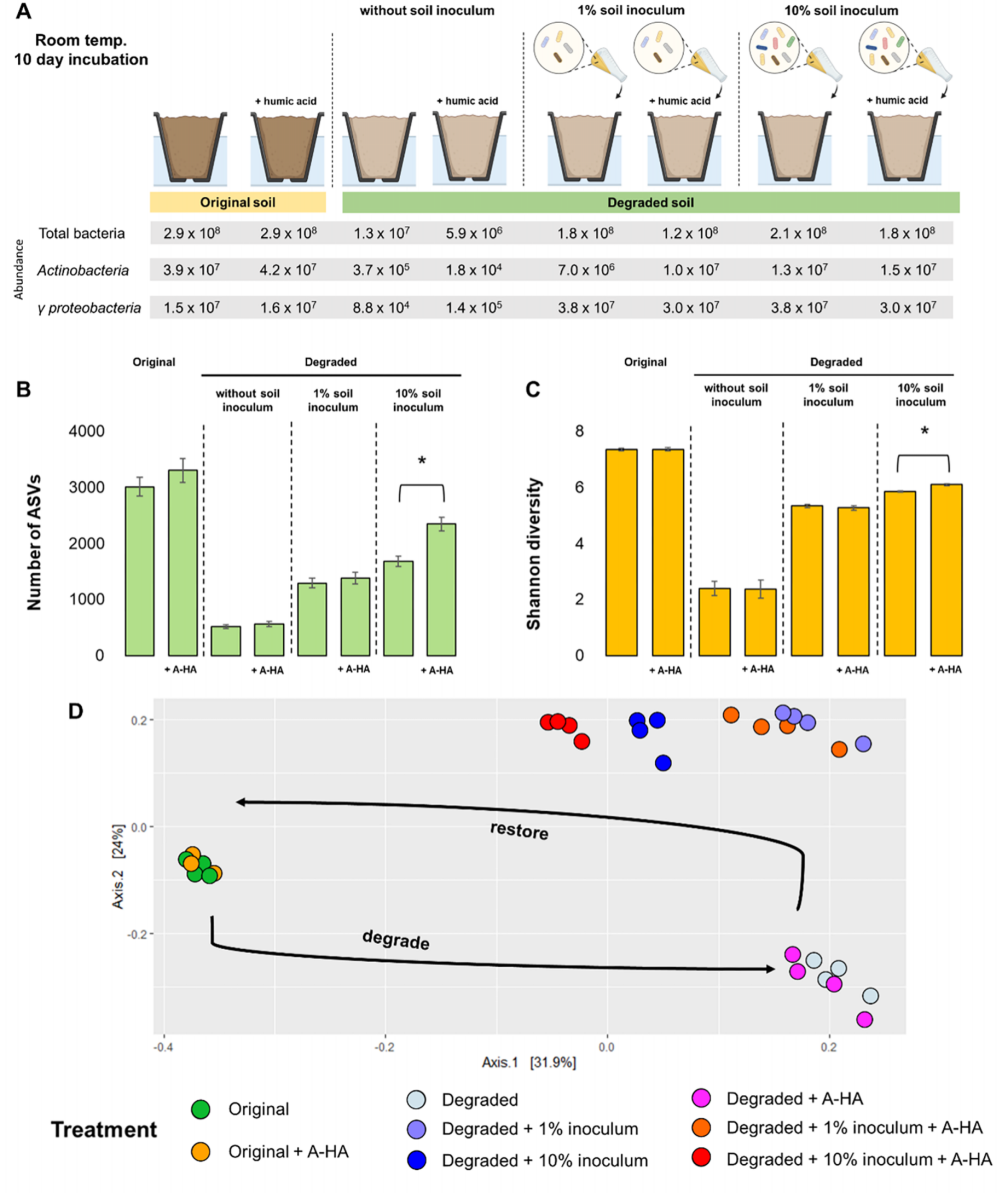
Abundance, bacterial richness, diversity, and community structure in the first experiment using soil RG. The abundances (16S rRNA gene copies per g of soil) of total bacteria, *Gammaproteobacteria*, and *Actinobacteria* were quantified using qPCR (A). Bacterial richness (B) and diversity (C) were estimated based on amplicon sequencing data. PCoA plots show the bacterial community structure of all samples (D). Bars marked with an asterisk indicate a statistically significant difference between treatments, based on post-hoc Dunn’s tests (*P* < 0.05).

A reduction of total bacteria, *Actinobacteria*, and *Gammaproteobacteria* 16S rRNA gene abundances was observed in degraded soil RG (1.3 × 10^7^ copies g^−1^, 3.7 × 10^5^ copies g^−1^, and 8.8 × 10^4^ copies g^−1^, respectively) in comparison to original soil RG (2.9 × 10^8^ copies g^−1^, 3.9 × 10^7^ copies g^−1^, and 1.5 × 10^7^ copies g^−1^, respectively). Number of total bacteria (ranged between 1.2 and 2.1 × 10^8^ copies g^−1^), *Actinobacteria* (ranged between 7.0 × 10^6^ copies g^−1–^1.5 × 10^7^ copies g^−1^), and *Gammaproteobacteria* (ranged between 3.0 and 3.8 × 10^7^ copies g^−1^) were higher in degraded soil with the addition of 1% and 10% soil transplants when compared to the degraded soil (Fig. 2A). These results indicated that soil transplants could restore bacterial abundance to the initial number. When comparing soil with and without humic acid treatments, no differences in total bacteria, *Actinobacteria*, and *Gammaproteobacteria* 16S rRNA gene abundances were observed (*P* > 0.05).

Soil transplants increased bacterial richness and diversity in the degraded soils. As anticipated, autoclaving soil significantly reduced bacterial richness. The bacterial richness (n_ASV_) decreased from 3 008 ASVs to 516 ASVs. Additionally, bacterial diversity, as measured by the Shannon index (H’), declined from 7.3 to 2.4. Degraded soil with 10% soil transplant had higher bacterial richness and diversity followed by degraded soil with 1% soil transplant, and degraded soil without soil transplant (*P* < 0.05, Fig. 2B and C). Interestingly, the application of humic acid significantly increased bacterial richness and Shannon diversity (*P* = 0.029 and *P* = 0.029, respectively) in degraded soil with the addition of 10% soil transplants (n_ASV_ = 2345 ± 246 and H’ = 6.1 ± 0.08) in comparison to the same soil but without the addition of humic acid (n_ASV_ = 1680 ± 190 and H’ = 5.8 ± 0.05, Fig. 2B and C). The observed trends indicate that the application of humic acid also increased bacterial richness in the original soil, degraded soil without soil inoculum, and degraded soil with 1% soil inoculum, but these differences were not statistically significant (Fig. 2B and C, *P* > 0.05).

When all of the factors were analyzed together, autoclaving and transplanting from the original soil were the main drivers of the bacterial community structure changes (*R*^2^ = 0.291, *P* = 0.001 and *R*^2^ = 0.287, *P* = 0.001), whereas humic acid addition only explained a small amount of the variation (*R*^2^ = 0.026, *P* = 0.071). The interaction between soil, the inoculum, and humic acid also explained a small amount of the variation (*R*^2^ = 0.051, *P* = 0.035). We observed that degraded soil with the addition of soil transplants clustered separately from original soil and degraded soil without soil transplants (Fig. 2D).

By analyzing separately the original and degraded soils, the humic acid application had a greater effect on changes in the bacterial community within the degraded soil with 10% soil transplants (*R*^2^ = 0.252, *P* = 0.030) in comparison to the degraded soil with 1% soil inoculum (*R*^2^ = 0.207, *P* = 0.038), and the degraded soil without soil transplants (*R*^2^ = 0.198, *P* = 0.026). Interestingly, the humic acid application in original soils explained less bacterial variation (*R*^2^ = 0.177, *P* = 0.023).

In our analysis of bacterial community similarity across various treatments relative to original soil, which served as the reference, we observed that the addition of 10% soil transplant resulted in a high microbial similarity, ranging from 42% to 67%, relative to the original state. In contrast, samples without soil transplant exhibited a lower similarity range of 8% to 25%. Moreover, we observed that the similarity was significantly higher in the degraded soil supplemented with humic acid compared to the soil that did not receive any humic acid amendment (Supplementary Fig. S1A). Altogether, the results show that humic acid likely has a stronger effect on bacterial richness and community structure in degraded soil than in the original soil.

### Experiment 2–The effect of humic acid depends on the native soil bacterial community composition

**Figure 3:**
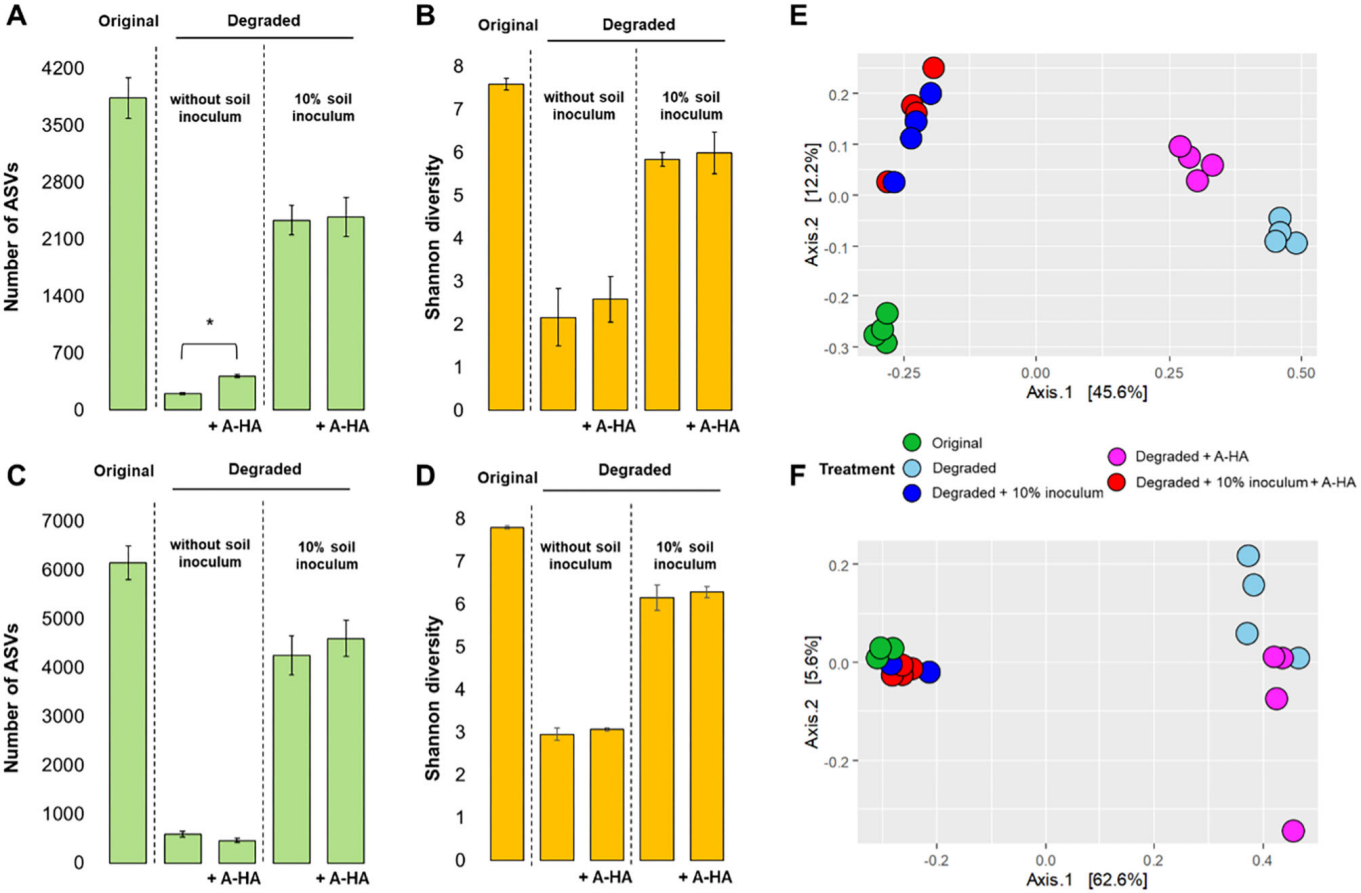
Bacterial richness, diversity, and community structure in the second experiment using soil EG and AO. Bacterial richness (A—soil EG and C—soil AO) and diversity (B—soil EG and D—soil AO) were estimated based on amplicon sequencing data. PCoA plots show bacterial community structure in soil EG (E) and soil AO (F). Bars marked with an asterisk indicate a statistically significant difference between treatments, based on post-hoc Dunn’s tests (*P* < 0.05).

As we observed that soil transplantation and the addition of humic acid increased bacterial diversity and influenced the composition of bacterial communities, we conducted a follow-up experiment using two contrasting soil types. This experiment aimed to assess whether humic acid treatment consistently affects bacterial community structure from two contrasting soil types. The soil’s origins (soil RG, soil EG, and soil AO) accounted for 82.1% of the variation in bacterial communities, indicating that the three soils utilized in our experiment harbor significantly distinct bacterial compositions (*P* = 0.001). Our analysis of bacterial community similarity indicated that the similarity between soil RG and soil EG (30.9%) was slightly higher than that between soil RG and soil AO (27.0%). As previously noted in Experiment 1, autoclaving soil significantly reduced bacterial richness and diversity. However, soil transplants partially restored bacterial richness and diversity (Fig. 3A–D). Soil transplantation was consistently identified as the primary factor affecting the composition of bacterial community structures (Fig. 3E and F). This factor explained 18.1% and 15.9% of bacterial community variations in soil EG and soil AO. Degraded soil with 10% soil inoculum was effective in restoring the bacterial community structure, as indicated by the samples aligning more closely with original soil, particularly in soil AO.

Humic acid did not significantly change the bacterial abundance, a pattern that was observed from the 1st experiment. Although it was not significant, a reduction of *Gammaproteobacteria* 16S rRNA gene abundances (2.8 × 10^4^ copies g^−1^ without soil inoculum and 1.5 × 10^7^ copies g^−1^ with 10% soil inoculum, Supplementary Fig. S2) was observed in degraded soil EG with humic acid treatments in comparison to degraded soil EG without humic acid treatments (5.2 × 10^4^ copies g^−1^ without soil inoculum and 1.9 × 10^7^ copies g^−1^ with 10% soil inoculum). Conversely, a distinct pattern in soil alpha diversity was observed. The abundances of *Gammaproteobacteria* 16S rRNA genes in degraded soil without humic acid treatment were higher than those in soil that received humic acid treatments.

Regarding the impact of the humic acid amendment on bacterial richness and diversity, the results obtained did not show consistent findings when compared to experiment 1. In both soil samples, the application of humic acid did not lead to a significant increase in bacterial richness or diversity in degraded soil with the addition of 10% soil transplants, when contrasted with the same soil that did not receive humic acid (Figs 3A–D). However, we noted an increasing trend in bacterial diversity was observed in the degraded soil EG with the addition of a soil transplant and A-HA (n_ASV_ = 2374 ± 487 and H’ = 6.0 ± 0.48), in contrast to the degraded soil that was not amended with humic acid (n_ASV_ = 2334 ± 363 and H’ = 5.8 ± 0.18). In our analysis of the bacterial community composition between different treatments relative to the original soil EG, we found that the similarity was significantly greater in the degraded soil EG supplemented with humic acid compared to the degraded soil EG that did not receive any humic acid amendment (Supplementary Fig. S1B). However, this pattern was not observed when humic acid was added to soil AO (Supplementary Fig. S1C).

### Enrichment of putative slow-growing bacteria through humic acid treatment

**Figure 4:**
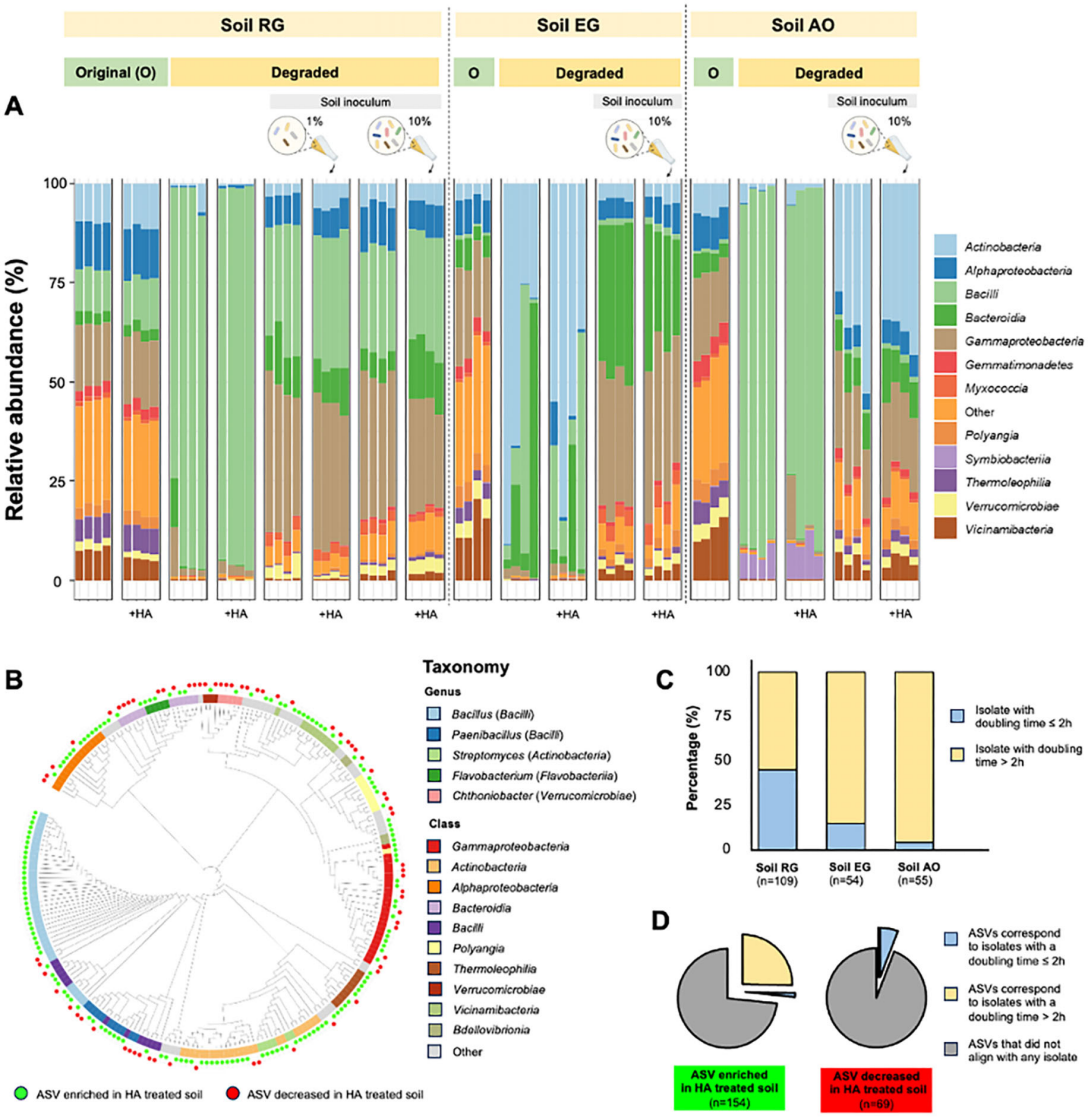
The composition of bacterial communities and the variations in taxa abundance in soil treated with humic acid compared to soil without humic acid amendment. Bacterial composition is shown at class level (A). A phylogenetic tree based on partial 16S rRNA genes of differentially abundant ASVs is illustrated (B). Different colors in the tree represent bacterial taxonomy at genus and class levels. ASVs with green dots were enriched in humic acid-treated soil, whereas ASVs with red dots were decreased in HA-treated soil according to the DeSeq2 analysis. Doubling time (h) of bacterial isolates cultivated from soil RG, EG, and AO (C). ASVs that exhibited enrichment and a reduction in soil treated with humic acid, in comparison to soil without the humic acid amendment, with a high similarity to the representative bacterial isolate 16S rRNA sequences (D).

Changes in bacterial community compositions due to soil transplants were observed (Fig. 4A). In the original soil, the predominant bacterial classes observed were *Gammaproteobacteria, Alphaproteobacteria, Vicinamibacteria, Actinobacteria*, and *Bacilli*. Following autoclaving, *Bacilli* was found to be the dominant bacterial class in the soil (soil RG: 88.4%; soil EG: 20.5%; soil AO: 89.4%). *Actinobacteria* were also significantly abundant in degraded soil EG, constituting 52.8% of the bacterial population. After the soil transplant, the relative abundance of *Gammaproteobacteria* increased in the degraded soil (soil RG: 35.9%; soil EG: 34.1%; soil AO: 21.3%, Fig. 3). Additionally, specific bacterial groups showed enrichment in each soil type; for instance, *Bacilli* (soil RG: 30.0%), *Bacteroidia* (soil EG: 37.6%), and *Actinobacteria* (soil AO: 38.1%), and surpassed the levels observed in the original soil.

We further investigated which bacterial taxa respond to humic acid treatment. The differential abundance using DeSeq2 was significant for 223 ASVs between soil treated with and without HA (Fig. 4B). A total of 154 bacterial ASVs were enriched in soil treated with humic acid, while 69 bacterial ASVs exhibited a reduction in abundance in the same treated soil. We observed a higher number of genera that belong to *Actinobacteria*, enriched in A-HA-treated soil (*n* = 21) compared to the soil without HA treatment (*n* = 3). A similar pattern was observed in the ASVs associated with *Alphaproteobacteria, Thermoleophilia*, and *Vicinamibacteria*. Notably, we also identified a clade of ASVs belonging to *Bacillus* that was enriched in HA-treated soil (Fig. 4B). By analyzing bacteria isolated from the same soil samples, we found that isolates (n = 218) with a fast-doubling time of less than 2 h were more prevalent in samples from soil RG (45%), followed by soil EG (14.9%) and soil AO (3.8%) (Fig. 4C). This indicates that HA treatment may be more effective in soils predominantly inhabited by fast-growing bacteria. By comparing the sequences from the isolates with the sequences of enriched ASVs in HA treated soil, we observed that a number of the enriched ASVs (25.3%, Fig. 4D) were highly similar to isolates with a slow doubling time of more than 2 h. Our results show that the HA-induced community shifts are likely to be due to the enrichment of slow-growing bacteria and/or the suppression of fast-growing bacteria.

### Water-extractable chemical compounds are depleted by inoculation with humic acid

**Figure 5:**
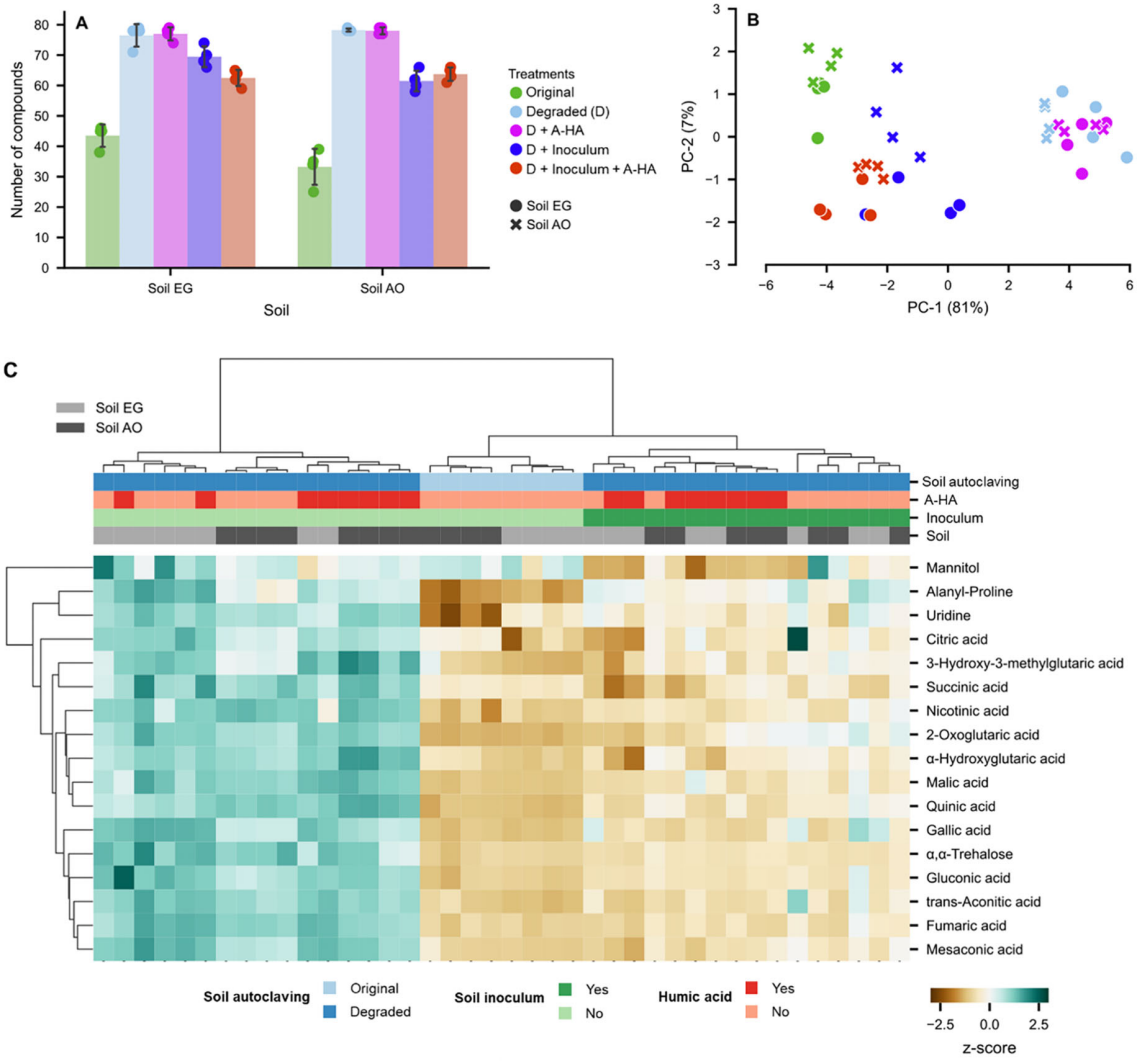
Number and composition of water-extractable compounds and mixtures. The number of compounds detected in soil using LC-MS (A). The principal component analysis (PCA) of the composition of water-extractable compounds in soil (B). The heat map illustrates the distribution of compounds that displayed differences between the samples (C).

The LC-MS measurements revealed that the treatments induced systematic changes in the water-extractable chemical compounds in the soils EG and AO (Fig. 5). As anticipated, autoclaving the soils substantially increased the total number of different compounds to 77 and 78 compared to the original soils, which had only 44 and 33 for soils EG and AO, respectively (Fig. 5A). The addition of HA to degraded soils did not alter the number of compounds considerably. However, providing an inoculum reduced the number of compounds to 70 and 62 for the two soils. Interestingly, the HA treatment together with the inoculum, compared with only the inoculum, had fewer compounds in soil EG but not soil AO (Fig. 5A). Overall, the water-extractable mixtures were very similar between the soils and responded similarly to the treatments (Fig. 5B). Furthermore, the difference between samples within each treatment was reduced with the addition of HA, indicating a common modification of the different chemical profiles. The shared compounds among all treatments and soils were mostly small organic acids (Fig. 5C) and the degraded treatments had most of the unique compounds for both soils (Supplementary Fig. S3). The drastic enrichment of water-extractable compounds after autoclaving offers a distinct metabolic profile that is altered by inoculation. The inoculated soils cluster closer to the original soils than to the degraded soils and share a similar metabolome except for a few compounds such as mannitol, which was more abundant in the original soils and was reduced in the degraded and inoculated treatments (Fig. 5C).

Comparing the compound composition of soil EG with and without humic acid amendments indicated that humic acid positively influenced the restoration of the degraded soil by bringing it closer to the original soil (Supplementary Fig. S4). However, this pattern was not observed in soil AO. Next, we focused on the enrichment or depletion of compounds in the presence of A-HA in degraded soils and compared their mean abundance to their mean abundance in the soils that did not receive A-HA. We observed mostly reductions of compounds by A-HA addition for both soils (Supplementary Fig. S5) while autoclaving increased the number of compounds (Fig. 5A). From the literature, we identified multiple important compounds that were substantially depleted, including precursors for metabolic activity (e.g. nicotinamide, 3′-AMP), readily degradable sugars (e.g. trehalose, hexose, mannitol), numerous small organic acids (e.g. succinic acid, furamic acid, citric acid), and a precursor of penicillium (4-hydroxybenzaldehyde). These compounds play important roles in cellular metabolism (production of ATP and NAD) and the survival of different taxa (production of antibiotics). Among the three compounds that were enriched after HA treatment, two were naturally occurring phenols, including acetosyringone (3′,5′-dimethoxy-4′-hydroxyacetophenone), which plays a role in plant-pathogen recognition (Baker et al. 2005), and phloretin, which can inhibit transport-mediated glucose uptake in human retinal cells (Hytti et al. 2023) and the bacterium *Escherichia coli* (Ma et al. 2022). Another compound that was enriched under A-HA treatment was pipecolic acid (a derivative of lysine catabolism), a non-proteogenic amino acid that confers immunity in plants and humans (Wang et al. 2018) and protects from osmotic stress in bacteria (Neshich et al. 2013).

## Discussion

In this study, we manipulated the soil microbial diversity through autoclaving and tested the hypothesis that soil transplants and humic acid amendment can restore microbial diversity to its original condition. Soil autoclaving promotes the proliferation of two bacterial taxa, specifically *Firmicutes* and *Gammaproteobacteria*, which are considered fast-growing copiotrophic bacteria (Ho et al. 2017, Stone et al. 2023). As expected, transplanting soil from the same soil type can facilitate the restoration of bacterial abundance and diversity, leading to a more balanced soil microbiome. Notably, a 10% soil inoculum was more effective in restoring abundance and diversity than a 1% soil inoculum. This aligns with the research of Howard et al. (2017), which indicated that a greater quantity of the transplanted original soil correlates with a more similar development of the microbiome in the new environment. Interestingly, the restoration of bacterial abundance and diversity in soils treated with the soil inocula was accompanied by the removal of many water-extractable compounds that were introduced by autoclaving. The observed differences were greater than what could be expected from the amended proportion of 10% soil inoculum. Hence, the observed restoration may have resulted from the activity and metabolite production of the transplanted microorganisms. Our results indicate that soil transplants can effectively restore depleted soil microbiomes and modify the soil nutrient profiles.

The combination of soil inoculum and humic acid led to significant alterations in bacterial community composition. It is important to note that humic acid resulted in a relatively minor alteration compared to soil inoculum treatment in soil compositions. The humic acid treatment was also dependent on the soil conditions. The utilization of humic acid has been found to enhance crop yields; however, its effectiveness is influenced by environmental and soil conditions, soil composition, and the types of crops being cultivated (Ampong et al. 2022). For example, the adsorption of humic acids onto clay surfaces is a key factor influencing their effectiveness (Lu et al. 2023). Soils with higher clay content provide more surface area for humic acids to interact, potentially enhancing their capacity to modify nutrient availability, which may in turn affect microbial composition, and vice versa. In soil RG with the highest clay content, humic acid addition significantly increased the alpha diversity in the degraded soil with 10% soil inoculum compared to without. However, this restorative effect could not be observed in the two other soils. This suggests that the soil properties i.e. clay content, including the microbial component must be considered to achieve optimal results when utilizing humic acid-based products for improving the soil microbiome and promoting plant growth. The addition of humic acid at concentrations up to 5% can significantly enhance species richness, whereas higher concentrations have no effect or even negative effect on soil bacterial diversity (Li et al. 2019). The authors suggested that rare taxa were the group that responded to the addition of humic acid, contributing to the increase in overall diversity. In contrast to the Shannon diversity index, the number of species gives greater weight to rare taxa (Chiarello et al. 2022). In our study, when comparing the number of ASVs (species) between treatments, the differences became more pronounced compared to Shannon diversity index values, suggesting that humic acids likely influence rare taxa. Interestingly, humic acid amendment affected the microbial community composition of the degraded soil making it more similar to the original soil. Overall, the soil transplants from healthy soils could be used as a potential treatment for microbially depleted soils and in combination with humic acid even greater effects could be achieved.

Humic acid likely decelerates the colonization of fast-growing copiotrophic bacteria in the soil. The relative abundance of *Gammaproteobacteria* showed a slight reduction due to humic acid treatment, indicating that humic acid could suppress the growth of *Gammaproteobacteria*, which are typically fast-growing (Kurm et al. 2017). The impact of humic acid was most pronounced in soil RG, followed by soil EG and AO. Notably, a greater number of bacterial isolates from soil RG exhibited a doubling time of less than 2 h compared to those from soil EG and AO. We hypothesize that the influence of humic acid on microbial diversity may be attributed to the relative abundance of fast- and slow-growing bacteria present in the soil. Additionally, the 16S rRNA amplicon sequence variant (ASV) analysis indicated that bacterial taxa enriched in the humic acid-treated soil exhibited a high similarity to 16S rRNA sequences of isolates with longer doubling times. Thus, our findings suggest that slow-growing taxa were likely enriched in the humic acid-treated soil. HA are chemically complex and slowly bioavailable, making them an unlikely primary carbon source for fast-growing, copiotrophic bacteria. HAs can also alter nutrient availability through sorption, complexation, and redox interactions (Zanin et al. 2019), providing a disadvantage for nutrient-demanding taxa while favoring slower-growing microorganisms adapted to low-nutrient conditions. The suppression of fast-growing bacteria may provide an opportunity for slow-growing bacteria to occupy their niches, proliferate, and ultimately contribute to an increase in overall bacterial diversity.

The integration of metabolomic and microbial datasets indicates that modifications in soil nutrient composition may mechanistically underlie the observed restructuring of bacterial communities. Autoclaved soil contained high amounts of in water-extractable organic compounds, including labile sugars and organic acids, which likely facilitated the rapid proliferation of copiotrophic taxa capable of utilizing readily available carbon sources. Conversely, the addition of humic acid reduced the levels of these labile compounds, such as trehalose, hexose, mannitol, and citric acid, thereby decreasing the pool of easily degradable substrates. By limiting the availability of nutrient-rich molecules and introducing more complex aromatic structures such as acetosyringone, phloretin, and pipecolic acid, humic acid appears to shift the metabolic environment toward conditions that favor slow-growing oligotrophic and functionally diverse microbial groups.

Despite the comprehensive analysis conducted, this study presents several important limitations. One key constraint lies in the method used to simulate soil degradation. The use of autoclaving, while effective for sterilization and microbial removal, does not realistically reflect natural or agricultural processes of soil degradation. In actual agronomic practice, comparable effects are achieved through methods such as steam sterilization or chemical fumigation. Therefore, future studies should aim to replicate soil degradation and recovery under more realistic field or greenhouse conditions to enhance ecological relevance. Additionally, the study focused primarily on the recovery of the soil bacterial diversity and structural composition in the degraded soil. While this provides valuable insights into community shifts, it does not account for the functional implications of these microbial changes. Incorporating measures of ecosystem functions—such as crop growth performance, organic matter decomposition, nutrient cycling, or enzyme activities—will offer a more comprehensive understanding of how microbial alterations influence soil health and agricultural productivity.

In conclusion, this study provides valuable insights into the restoration of soil microbiomes through the application of soil inoculum. The effectiveness of soil transplants may be enhanced using humic substances, which facilitate the recovery of slow-growing bacteria. Further research investigating the effects of A-HAs at varying concentrations across different soil types will deepen our understanding of its impact on soil microbiomes and their ecological traits. Addressing environmental challenges that disrupt soil microbiomes is increasingly important and exploring sustainable solutions, such as utilizing A-HAs and soil transplants from healthy soils, may serve as effective strategies for restoring soil health worldwide.

## Supplementary Material

qvag012_Supplemental_File

## Data Availability

The sequencing data has been deposited in the European Nucleotide Archive (ENA) database under the study number PRJEB75611.
