# Supplementary material for: Microbiome interventions combined with artificial humic acid treatments for restoring soil bacterial diversity
Source: Sustain Microbiol. 2026 Mar 18;3(2):qvag012. doi: 10.1093/sumbio/qvag012 (PMC13375291; doi:10.1093/sumbio/qvag012)
Supplement: Supplementary file 1 [file qvag012_Supplemental_File.docx]

**Supplementary Materials**

**Supplementary Tables**

**Table S1 Physico-chemical properties of the three soils used**

| **Soil** | **Sand (%)** | **Silt (%)** | **Clay (%)** |  |  |  |  |  |  |
| --- | --- | --- | --- | --- | --- | --- | --- | --- | --- |
| RG | 24.8 | 55.8 | 19.4 |  |  |  |  |  |  |
| EG | 35 | 54.7 | 10.3 |  |  |  |  |  |  |
| AO | 37.7 | 54.9 | 7.4 |  |  |  |  |  |  |
|  |  |  |  |  |  |  |  |  |  |
| **Soil** | **pH** | **K (mg/kg)** | **P (mg/kg)** | **Organic matter (%)** |  |  |  |  |  |
| RG | 6.9 | 196 | 40 | 3.4 |  |  |  |  |  |
| EG | 7.2 | 40 | 357 | 5.5 |  |  |  |  |  |
| AO | 7.1 | 63 | 202 | 5.1 |  |  |  |  |  |
|  |  |  |  |  |  |  |  |  |  |
| **Soil** | **C/N** | **Ca (cmolc/kg)** | **Mg (cmolc/kg)** | **K (cmolc/kg)** | **Na (cmolc/kg)** | **Al (cmolc/kg)** | **Mn (cmolc/kg)** | **H (cmolc/kg)** | **CEC (cmolc/kg)** |
| RG | 9.08 | 16.13 | 2.14 | 0.62 | <0.04 | <0.06 | 0.29 | 0.002 | 19.21 |
| EG | 11.63 | 29.05 | 1.57 | <0.20 | <0.04 | <0.06 | 0.01 | 0.002 | 30.77 |
| AO | 11.26 | 21.69 | 2.94 | <0.20 | <0.04 | <0.06 | <0.01 | 0.002 | 24.83 |

**Supplementary Figures**

**Supplementary Figure S1. Bacterial community similarity between different treatments compared the original soil.** Pairwise Bray-Curtis distances were calculated to assess the overall variation in soil microbiome across different treatments.

**Supplementary Figure S2.** The abundances (16S rRNA gene copies^-1^) of total bacteria (A – Soil EG and D – Soil AO), *Actinobacteria* (B – Soil EG and E – Soil AO) and *Gammaproteobacteria* (C – Soil EG and F – Soil AO) were quantified using qPCR (A).

**Supplementary Figure S3. Number of unique chemical compounds per treatment for two soils**.

**Supplementary Figure S4. Soil compound profile similarity between different treatments compared to non-autoclaved soil.** Soil origin - Soil EG (A) and Soil AO (B). Pairwise Euclidean dissimilarities were calculated to assess variation in soil compound composition among samples subjected to different treatments.

**Supplementary Figure S5. Mean log-response ratio of humic acid amendment.** Whiskers show the standard error (SE). Only compounds are shown which are at least two SE away from zero**.**
